# Protocol-directed weaning *versus* conventional weaning from mechanical ventilation for neurocritical patients in an intensive care unit: a nonrandomized quasi-experimental study

**DOI:** 10.5935/2965-2774.20230340-en

**Published:** 2023

**Authors:** Alberto Belenguer-Muncharaz, Carmen Díaz-Tormo, Estefania Granero-Gasamans, Maria-Lidón Mateu-Campos

**Affiliations:** 1 Intensive Care Unit, Hospital General Universitari de Castelló - Castelló, Spain

**Keywords:** Critical illness, Airway extubation, Weaning, Ventilator weaning, Tracheotomy, Respiration, artificial

## Abstract

**Objective:**

To investigate whether protocol-directed weaning in neurocritical patients would reduce the rate of extubation failure (as a primary outcome) and the associated complications (as a secondary outcome) compared with conventional weaning.

**Methods:**

A quasi-experimental study was conducted in a medical-surgical intensive care unit from January 2016 to December 2018. Patients aged 18 years or older with an acute neurological disease who were on mechanical ventilation > 24 hours were included. All patients included in the study were ready to wean, with no or minimal sedation, Glasgow coma score ≥ 9, spontaneous ventilatory stimulus, noradrenaline ≤ 0.2µgr/kg/ minute, fraction of inspired oxygen ≤ 0.5, positive end-expiratory pressure ≤ 5cmH_2_O, maximal inspiratory pressure < -20cmH_2_O, and occlusion pressure < 6cmH_2_O.

**Results:**

Ninety-four of 314 patients admitted to the intensive care unit were included (50 in the Intervention Group and 44 in the Control Group). There was no significant difference in spontaneous breathing trial failure (18% in the Intervention Group *versus* 34% in the Control Group, p = 0.12). More patients in the Intervention Group were extubated than in the Control Group (100% *versus* 79%, p = 0.01). The rate of extubation failure was not signifiantly diffrent between the groups (18% in the Intervention Group *versus* 17% in the Control Group; relative risk 1.02; 95%CI 0.64 - 1.61; p = 1.00). The reintubation rate was lower in the Control Group (16% in the Intervention Group *versus* 11% in the Control Group; relative risk 1.15; 95%CI 0.74 - 1.82; p = 0.75). The need for tracheotomy was lower in the Intervention Group [4 (8%) *versus* 11 (25%) in the Control Group; relative risk 0.32; 95%CI 0.11 - 0.93; p = 0.04]. At Day 28, the patients in the Intervention Group had more ventilator-free days than those in the Control Group [28 (26 - 28) days *versus* 26 (19 - 28) days; p = 0.01]. The total duration of mechanical ventilation was shorter in the Intervention Group than in the Control Group [5 (2 - 13) days *versus* 9 (3 - 22) days; p = 0.01]. There were no diffrences in the length of intensive care unit stay, 28-day free from mechanical ventilation, hospital stay or 90-day mortality.

**Conclusion:**

Considering the limitations of our study, the application of a weaning protocol for neurocritical patients led to a high percentage of extubation, a reduced need for tracheotomy and a shortened duration of mechanical ventilation. However, there was no reduction in extubation failure or the 28-day free of from mechanical ventilation compared with the Control Group.

**ClinicalTrials.gov Registry:** NCT03128086

## INTRODUCTION

Many neurocritical patients require invasive mechanical ventilation (MV) to protect the airway, provide adequate oxygenation and prevent aspiration.^(1,2)^ The appropriate level of consciousness needed to advance the process of weaning from MV when patients with brain injuries improve is unclear, thereby delaying the onset of weaning. Although it is recommended in the guidelines that a higher level of consciousness is needed to start the process of weaning from MV, there is no cutoff point.^(3,4)^ Therefore, delayed extubation has been related to prolonged MV, a longer intensive care unit (ICU) stay and higher rates of pneumonia and mortality.^(5,6)^ Additionally, the rate of extubation failure in neurocritical patients (e.g., dysfunctional airway reflexes, prolonged sedation, and reduced pharyngeal tone) ranges between 10 and 35%. ^(2,7,8,9,10)^ Extubation failure is associated with prolonged M V, a longer ICU stay, and an increased risk of infection and mortality.^(5,6,11,12)^ Therefore, extubation in neurocritical patients is a challenge; both early and delayed extubation are accompanied by a risk of complications.

In nonneurocritical patients, a spontaneous breathing trial (SBT) is usually performed before extubation to assess the patient’s ability to breathe spontaneously.^(3,13)^ However, in neurocritical patients, a successful SBT cannot be used to determine if extubation will be successful. Several studies have shown high extubation failure rates despite a previously successful SBT.^(6,7,9,10)^ After a successful SBT, the capacity to maintain a patent airway and the parameters used to analyze respiratory muscle strength to guarantee successful extubation should be assessed.^(7,10,14,15,16,17,18,19)^

Use of a weaning protocol for nonneurocritical patients reduced the duration of MV and the length of ICU stay compared to the physicians’ decisions.^(3,20)^ A meta-analysis showed that weaning protocols reduced the duration of MV, weaning time and length of ICU stay. ^(21)^ Unfortunately, the results for neurocritical patients are inconclusive,^(12,22)^ with the exception of two studies that demonstrated a benefit of the application of the weaning protocol in neurological patients.^(23,24)^

In the present study, we investigated whether protocol-directed weaning would reduce the rate of extubation failure and the associated complications in neurocritical patients compared to conventional weaning. The primary objective was the rate of extubation failure. As secondary objectives, we evaluated SBT failure; extubation and reintubation rates; incidence of complications (infections, acute renal failure); need for tracheotomy; duration of M V, ICU and hospital length of stay; and mortality in the ICU, in the hospital and at 90 days.

## METHODS

### Study population

A quasi-experimental study was conducted in a medical surgical ICU from January 2016 to December 2018.^(25)^ The study protocol was approved by the Clinical Research Ethics Committee of the *Hospital General Universitari de Castelló* (number 10/2015). Written informed consent was obtained from legal representatives of the patients who were included in the study. The study was registered on ClinicalTrials.gov (NCT 03128086). Patients aged 18 years or older with an acute medical or surgical neurological disease (acute ischemic or hemorrhagic stroke, acute subarachnoid hemorrhage, traumatic brain damage, metabolic encephalopathy - toxic or infectious, scheduled neurosurgery with prolonged MV > 24 hours, and status epilepticus) were included. For inclusion in the study, all patients undergoing MV needed to meet the following conditions:^(3)^ no or minimal sedation (propofol ≤ 1mg/kg/h or midazolam ≤ 0.1mg/ kg/h), spontaneous ventilatory stimulus, intracranial pressure < 20mmHg for 48 - 72 hours, Glasgow coma score (GCS) ≥ 9 (motor > 4 points),^(2,7,22,24)^ noradrenaline ≤ 0.2µgr/kg/minute, fraction of inspired oxygen (FiO_2_) ≤ 0.5, positive end-expiratory pressure (PEEP) ≤ 5cmH_2_O, no scheduled intervention in the subsequent 48 hours, maximal inspiratory pressure (MIP) < -20cmH_2_O,^(3,7)^ and occlusion pressure (P_0.1_) < 6cmH_2_O.^(3,7)^ The MIP and P_0.1_ values were obtained while the patient was under pressure-support ventilation (PSV) of 7cmH_2_O and 0cmH_2_O of PEEP for 2 minutes through the software available in Evita ventilators (Dräger, Germany).^(3,7,26)^ The exclusion criteria were as follows: scheduled neurosurgery (MV < 24 hours), neuromuscular disease, spinal cord injury, tracheotomized patients, patients who were not assessed to be ready to wean, MIP > -20cmH_2_O,^(3,7,26)^ P_0.1_ > 6cmH_2_O,^(7,26)^ severe multiple traumatic injuries, direct extubation or self-extubation, patients who died in the ICU under MV before the start of weaning and patients with do not reintubate orders.

**Figure 1:**
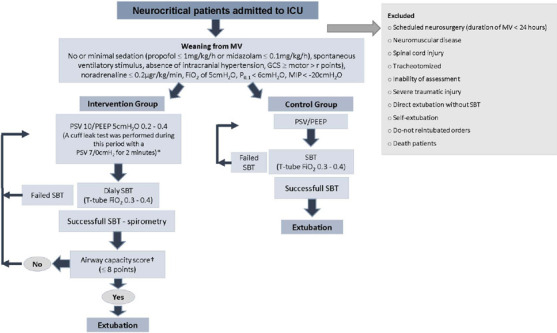
Weaning protocol

This quasi-experimental design of nonequivalent groups that include a Control Group and a pretest might be suitable to compare a weaning protocol (Intervention Group) *versus* conventional weaning (Control Group) in neurocritical patients and might reduce the likelihood of biases in this type of study.^(25)^ First, all patients included in the study met the criteria to start weaning (inclusion conditions). All inclusion conditions were assessed daily by a physician who did not participate in the weaning attempt. The attending physicians were blinded to several measurements (MIP and P_0.1_). Patients who did not meet the inclusion criteria or were not assessed by the investigators for inclusion in the study were excluded (Figure 1). Second, although these are nonequivalent groups (there was no randomization), the pretest comparison between intervention and Control Groups allowed us to assess the initial comparability of the groups and therefore increases the validity. Finally, the chosen weaning method (intervention *versus* control) and extubation were made at the discretion of the attending physician. Selection bias existed when the assignment depended on the physician’s decision. To avoid bias, the study must meet standards that ensure the reliability of the data obtained and the quality of the conclusions that can be drawn from them. Therefore, the study complied with the TREND statement (EQUATOR: https://www.equator-network.org/reporting-guidelines/improving-the-reporting-quality-of-nonrandomized-evaluations-of-behavioral-and-public-health-interventions-the-trend-statement).^(25)^

ICU - intensive care unit; MV - mechanical ventilation; SBT - spontaneous breathing trial; GCS - Glasgow coma score; FiO_2_ - fraction of inspired oxygen; P_0.1_ - occlusion pressure; MIP - maximum inspiratory pressure; PSV - pressure support ventilation; PEEP - positive end-expiratory pressure. * Performed in the last included patients (n = 16); † Airway capacity score: Number of aspirations/shift (none, 0; 1 asp, 1; 2 asp, 2; ≥ 3 asp, 3); cough capacity (strong, 0; mild, 1; weak, 2; absent, 3); gag reflex (strong: 0; moderate: 1; weak: 2; absent: 3); appearance of secretions, including viscosity (liquid: 0; frothy: 1; thick: 2; dry: 3) and color (clear: 0; brown: 1; yellow: 2; green: 3).

### Protocol study (Intervention Group)

The patients were ventilated in PSV (Figure 1), which was gradually reduced (until reaching a PSV of 10cmH_2_O above 5cmH_2_O of PEEP).^(24)^ In the last sixteen patients of the study, a cuff leak test was performed with a PSV of 7cmH_2_O and 0cmH_2_O of PEEP before the SBT because three patients with postextubation stridor were observed. Then, the patient was disconnected from the ventilator to a T-tube (SBT), which was considered the onset of the weaning attempt.^(3,7,10,14,19,22,27)^ All patients underwent daily SBT until they were extubated. Hemodynamic parameters (mean blood pressure - MBP, heart rate - HR), respiratory parameters (respiratory rate - RR, partial pressure of oxygen - PaO_2_, partial pressure of carbon dioxide - PaCO_2_, PaO_2_/FiO_2_ ratio and pH - through blood gas analysis, and transcutaneous oxygen saturation - SaO_2_), and neurological parameters (mean GCS) were collected at the beginning (5 minutes) and the end (between 30 and 120 minutes) of a successful SBT.^(28)^ An SBT was considered failed when more than 2 of the following criteria were observed: PaO_2_ < 50 - 60mmHg with FiO_2_ ≤ 0.5 or SaO_2_ < 90%, PaCO_2_ > 50mmHg, pH < 7.35, RR > 35bpm, HR > 140bpm, systolic blood pressure (SBP) > 180mmHg, cardiac arrhythmias, dyspnea, and increased use of accessory muscles.^(3)^ If the patient failed the SBT, they were reconnected to MV. A successful SBT was defined as the absence of any of the variables previously mentioned. Spirometry was performed using a Wright spirometer attached to a T-tube at the end of a successful SBT. The following parameters were collected: tidal volume (V_T_), RR, minute volume and calculated RR/V_T_ ratio.^(3)^

### Airway clearance capacity

After a successful SBT, airway capacity was evaluated by the following variables (Figure 1): number of aspirations of secretions/8-h nursing shift (none, 0; 1 asp, 1; 2 asp, 2; ≥ 3 asp, 3); appearance of secretions, including viscosity (liquid, 0; frothy, 1; thick, 2; dry, 3) and color (clear 0; brown, 1; yellow, 2; green, 3); cough capacity (strong, 0; mild, 1; weak, 2; absent, 3); and gag reflex after aspiration by nurses and evaluated by the attending physician (strong, 0; moderate, 1; weak, 2; absent, 3). A score ≤ 8 was considered adequate to maintain the permeability of the airway.^(6)^ Then, the endotracheal tube was removed, and the patient received oxygen through a Venturi mask (FiO_2_ of 0.3 - 0.4).^(19,27,28)^ If the score was > 8, the patient was connected to MV.

### Conventional weaning (Control Group)

Patients were weaned from MV (Figure 1) according to the usual procedure in our unit by reducing the level of PSV.^(19)^ Then, the patient was connected to a T-tube (the same hemodynamic and respiratory parameters as those used in the Intervention Group were collected). If the patient failed the SBT, they were reconnected to M V. ^(3)^ After a successful SBT, the patient was extubated and received conventional oxygen therapy.^(19,27,28)^

The investigators considered that all patients met the criteria and were ready to wean. In both groups, the attending physician decided when to start the SBT. In the Intervention Group, an SBT was performed followed by a series of measurements (e.g., cuff leak test, spirometry, airway clearance capacity) and then extubation of the patient. In the Control Group, an SBT was also performed, followed by extubation according to the subjective decision of the physician (based on level of consciousness, amount of secretions and ability to cough). The final decision to extubate or to be tracheotomized was left to the discretion of the responsible physician. In our unit, although there was no protocol, the patient might be tracheotomized according to the following criteria: prolonged MV (established at 21 days), a low level of consciousness after removal of sedation (GCS < 9), excess secretions and a failed SBT or extubation.^(19)^

Successful weaning was considered when a patient was extubated and did not need ventilatory support within 48 hours after extubation. Weaning failure was defined as failure of SBT; need for urgent reintubation (i.e., cardiac or respiratory arrest, neurologic deterioration, hemodynamic instability) or need for ventilatory support; or death within 48 hours following extubation.^(3,23)^ A patient who showed acute respiratory failure within 48 hours after extubation (use of accessory muscles, paradoxical breathing, RR > for 2 hours, HR > 140bpm, SaO_2_ < 90% or PaO_2_ < 80mmHg with FiO_2_ ≥ 0.5 or PaCO_2_ > 45mmHg) was considered an extubation failure, and the patient needed ventilatory support.^(3,7,12,14)^ According to previous studies, a neurocritical patient who fails extubation should be intubated.^(3,6,7,8,9,10,12,23)^ Based on our experience and publications, the use of noninvasive ventilation (NIV) after a failed extubation was not indicated for these patients, but a trial of NIV was left to the discretion of the attending physician.^(3,19,23,29)^ NIV or high-flow oxygen therapy was not considered an indication for the prevention of failed extubation. Similarly, the use of bronchodilators, aspiration of secretions and respiratory physiotherapy were left to the discretion of the attending physician and nurses.^(19)^

At ICU admission, the following variables were collected: age, sex, body mass index, comorbidities, Simplified Acute Physiological Score (SAPS) 3 for severity prognosis, Sequential Organ Failure Assessment (SOFA) for organ failure (at ICU admission), reason for MV, and GCS at the time of intubation. During the ICU stay, all treatments and neurological procedures were registered. The duration and types of sedatives and analgesics used until the first SBT were documented. Additionally, the time from the onset of MV to the first SBT and the time from the patient’s readiness to wean assessment to the first SBT were recorded. The duration of the last SBT was also measured.

After the first weaning attempt, the causes and rate of extubation failure, use of NIV, and need for reintubation were registered. The following complications during the ICU stay were recorded: the need for tracheotomy, infections (ventilator-associated pneumonia or tracheobronchitis, urinary tract infection, and bacteremia),^(30)^ the development of acute renal failure (and the need for continuous renal replacement therapy), ventilator-free status at 28 days and total duration of MV. Ventilator-free days were defined as the number of days, from Day 1 to Day 28, that a patient breathed spontaneously and was alive. Day 0 was defined as the day the patient met the criteria to start weaning. Total MV was defined as the duration of MV until the ventilator was finally switched off, which was defined as a definitive extubation or tracheotomy without the need for MV or NIV.

### Statistical analysis

Based on the previous results of our retrospective study (in which extubation failure was 26%),^(19)^ we considered that the extubation failure rate could be reduced by 13%^(3)^ (26% in the Control Group *versus* 13% in the Intervention Group). The estimated sample size for each group was 109 patients, with a confidence interval (1-α) = 95% (p = 0.05) and power (1-β) = 80%. The study was discontinued after 94 patients were included because some investigators moved to another hospital.

A comparative analysis of the quantitative variables, either parametric or nonparametric, was conducted using Student’s t test or the Mann‒Whitney U test. For the qualitative variables, we used the chi-square test or Fisher’s exact test. The relative risk and 95% confidence interval were calculated for the qualitative parameters to compare the effect of groups. The number of patients who were alive and breathing without MV at 28 days was analyzed by Kaplan-Meier curves (log rank test). The cumulative probability of 90-d survival was determined by Kaplan‒ Meier curves and the Breslow test. A time-dependent covariate was used to assume the proportionality of hazard ratios, with the aim of studying 90-d mortality.^(31)^ Statistical significance was reached if p < 0.05. The data were analyzed by using the Statistical Package for the Social Sciences (SPSS), version 22.0.

## RESULTS

**Figure 2:**
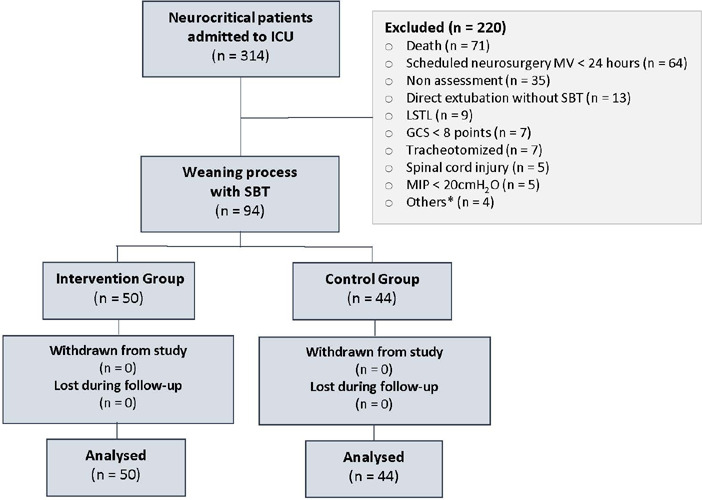
Study flowchart

During the study period, 94 of 314 patients admitted to the ICU (Figure 2) were included in the study (50 patients in the Intervention Group and 44 patients in the Control Group). As shown in table 1 and considering the limited number of patients and lack of randomization, there were no statistically significant differences in the baseline variables. Similarly, there were no significant differences in the sedative and analgesic drugs used or the complications and procedures performed during their ICU stay.

ICU - intensive care unit; MV - mechanical ventilation; SBT - spontaneous breathing trial; LSTL - life support therapy limitation; GCS - Glasgow coma score; MIP - maximal inspiratory pressure. * Neuromuscular disease (n = 2), self-extubation (n = 1), severe traumatic injury (n = 1).

**Table 1:** Baseline demographic characteristics, comorbidities, neurological diseases, level of consciousness at onset of mechanical ventilation, sedatives and analgesics employed, and intensive care unit interventions and complications

	Intervention Group (n = 50)	Control Group (n = 44)	p value
Gender, male	30 (60)	27 (61)	1.00*
Age (years)	54 ± 19	58 ± 19	0.34
BMI (kg/m^2^)	27 ± 4	29 ± 7	0.22
SAPS 3 at ICU admission	55 ± 15	53 ± 17	0.57
SOFA at ICU admission	6 ± 2	7 ± 3	0.1
Hypertension	16 (32)	16 (36)	0.67*
Diabetes mellitus	6 (12)	9 (20)	0.39*
Chronic obstructive pulmonary disease	2 (4)	5 (11)	0.24*
Chronic renal failure	1 (2)	4 (9)	0.17*
Ischemic heart disease	3 (6)	3 (7)	1.00*
Smoking	6 (12)	4 (9)	0.75*
Alcohol	2 (4)	3 (7)	0.65*
Neurologic disease			
Acute hemorrhagic stroke	15 (30)	12 (27)	
Traumatic brain injury	10 (20)	14 (32)	
Acute ischemic stroke	7 (14)	3 (7)	
Metabolic coma	8 (16)	8 (18)	0.52
Subarachnoid hemorrhage	6 (12)	3 (7)	
Status epilepticus	2 (4)	4 (9)	
Scheduled neurosurgical surgery MV > 24 hours	2 (4)	0 (0)	
Medical patient	40 (80)	32 (73)	0.46*
Surgical patient	10 (20)	12 (27)	
Characteristics of intubation			
Setting			
Outside of hospital	16 (32)	13 (29)	
Emergency department	12 (24)	11 (21)	
ICU	12 (24)	12 (27)	0.97
Other hospital	5 (10)	5 (11)	
Operating room	5 (10)	3 (7)	
Type of intubation			
Urgent intubation	44 (88)	40 (91)	0.07
Programmed intubation	6 (12)	4 (9)	
GCS (points) at ICU admission			
3 - 8	32 (64)	35 (79)	
> 8 - 12	10 (20)	6 (14)	0.21
> 12 - 15	8 (16)	3 (7)	
Sedation			
Propofol	29 (58)	19 (43)	
Propofol and midazolam	17 (34)	14 (32)	0.06*
Midazolam	0 (0)	5 (11)	
No sedation	4 (8)	6 (14)	
Analgesia†			
Morphine	34 (68)	34 (77)	
Fentanyl	5 (10)	1 (2)	0.46
Morphine and fentanyl	2 (5)	2 (4)	
No analgesia	9 (18)	7 (16)	
Duration of sedation (hours)	55 (31 - 93)	49 (25 - 81)	0.37
Duration of analgesia (hours)	73 (31 - 117)	75 (29 - 123)	0.95
Evolution at ICU			
Intracranial hypertension	8 (16)	3 (7)	0.2*
Decompressive craniectomy	2 (4)	2 (4)	1.00*
External ventricular drainage	6 (12)	8 (18)	0.56*

BMI - body mass index; SAPS - Simplified Acute Physiological Score; ICU - intensive care unit; SOFA - Sequential Organ Failure Assessment; MV - mechanical ventilation; GCS - Glasgow coma score. Results expressed as n (%), mean ± standard deviation or median and interquartile range (25 - 75). * Fisher test; † Time of use until first attempted extubation.

During the weaning period (Table 2), both the onset of MV and the time when the patient met the weaning criteria until the first SBT were shorter in the Intervention Group than in the Control Group [3 (1 - 5) days *versus* 5 (2 - 11) days; p = 0.01] and [8 (1 - 25) hours *versus* 23 (8 - 74) hours; p = 0.003]. Comparing both groups, there was no significant difference in the failure rate of SBT. The patients in the Intervention Group passed all the tests before extubation. At the end of the last SBT, there were no neurologic, hemodynamic, or respiratory differences between the two groups. After the first weaning attempt, more patients in the Intervention Group were extubated than in the Control Group (100% *versus* 79%; p = 0.01). Nine (20%) patients in the Control Group were directly (primary) tracheotomized.

**Table 2:** Respiratory parameters during pressure support ventilation and the last spontaneous breathing trial and outcome

	Intervention Group (n = 50)	Control Group (n = 44)	p value
Onset of MV - first SBT (days)	3 (1 - 5)	5 (2 - 11)	0.01
Criteria readiness to wean- first SBT (hours)	8 (1 - 25)	23 (8 - 74)	0.003
Respiratory parameters during of pressure support ventilation			
MIP* (cmH_2_O)	- 28 ± 6	- 31 ± 9	0.23
P_0.1_* (cmH_2_O)	5 ± 10	3 ± 2	0.56
Cuff leak test (negative result)	16/16 (100)	0	NA
Spirometry at the end of SBT†			
RR/VT (bpm/L)	59 ± 28	0	NA
Minute volume (L/minute)	10 ± 2.8	0	NA
Airway clearance capacity score	4 ± 2	0	NA
SBT failure	9 (18)	12 (34)	0.12
Respiratory measurement at the end of successful SBT			
GCS‡	9 ± 2	10 ± 1	0.08
Median blood pressure (mmHg)	102 ± 13	99 ± 13	0.47
Heart rate (bpm)	91 ± 20	83 ± 17	0.13
Respiratory rate (bpm)	24 ± 6	21 ± 7	0.19
pH	7.41 ± 0.13	7.43 ± 0.05	0.61
PaCO_2_, (mmHg)	37 ± 5	39 ± 6	0.44
PaO_2_/FiO_2_, (mmHg)	230 ± 93	254 ± 107	0.46
Duration of SBT* (minutes)	80 (60 - 157)	90 (60 - 180)	0.62
Outcome of first weaning attempt			
Direct tracheotomy	0 (0)	9 (20)§	0.001¶
Extubation	50 (100)	35 (79)	

MV - mechanical ventilation; SBT - spontaneous breathing trial; MIP - maximal inspiratory pressure; P_0.1_ - occlusion pressure at 100 ms; NA - not appropriate; RR/VT - respiratory rate to tidal volume ratio; GCS - Glasgow coma score; PaCO_2_ - partial pressure of carbon dioxide; PaO_2_/FiO_2_ - partial arterial oxygen pressure to inspired fraction of oxygen ratio. Results expressed as mean ± (standard deviation) or median (interquartile range) or n (%).* Performed in all patients to be included in the study, only recorded in 80 patients. †n = 41 patients in study group; ‡ the Glasgow coma score at the end of spontaneous breathing trial was assessed on the basis of the motor and ocular response items. Verbal response was considered 1 point because all the patients were intubated; § Causes of tracheotomy: low level of consciousness (n = 6), prolonged mechanical ventilation (n = 3): amount of secretions (n = 2), spontaneous breathing trial failure (n = 1). ¶ Fisher’s exact test.

**Figure 3:**
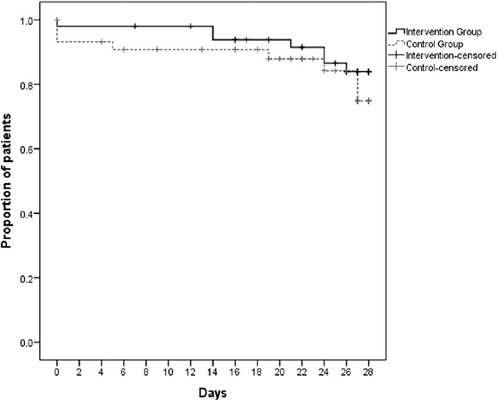
Number of ventilator-free days at Day 28

**Figure 4:**
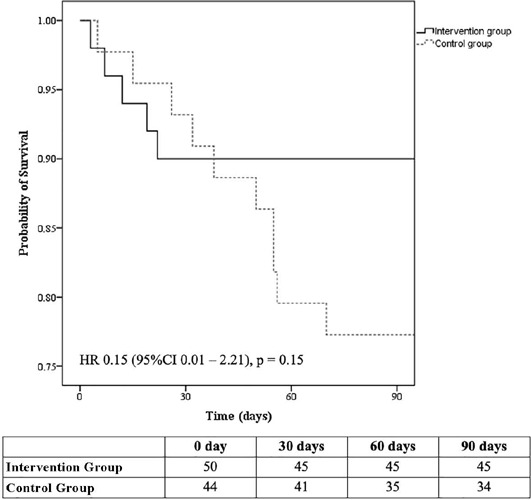
Kaplan-Meier **s**urvival analysis (with Breslow test) comparing the Intervention Group *versus* the Control Group after 90 days

Regarding the primary objective (Table 3), the extubation failure rate was not significantly different between the two groups (18% in the Intervention Group *versus* 17% in the Control Group; relative risk 1.02; 95%CI 0.64 - 1.61; p = 1.00). After applying NIV, the reintubation rate was lower in the Control Group (16% in the Intervention Group *versus* 11% in the Control Group; relative risk 1.15 0.74 - 1.82; p = 0.75). The number of patients who needed a tracheotomy was lower in the Intervention Group than in the Control Group [4 (8%) *versus* 11 (25%) in the Control Group; relative risk 0.32; 95%CI 0.11 - 0.93; p = 0.04]. There were no statistically significant differences among the remaining variables. At Day 28, the patients in the Intervention Group had more ventilator-free days than the Control Group [28 (26 - 28) days *versus* 26 (19 - 28) days; p = 0.01]. However, the analysis of the 28-d ventilation-free rate showed no differences between the two groups (Figure 3). The total MV time was shorter in the Intervention Group than in the Control Group [5 (2 - 13) days *versus* 9 (3 - 22) days; p = 0.01]. There were no differences in the length of ICU or hospital stay or 90-d mortality (Table 3 and Figure 4).

**Table 3:** Outcomes comparing both groups

	Intervention Group (n = 50)	Control Group (n = 44)	p value	Relative risk (95%CI)
Extubation failure* (n = 85)	9/50 (18)	6/35 (17)	1.00†	1.02 (0.64 - 1.61)
NIV after extubation failure (n = 85)	1/50 (2)	2/35 (6)	0.56†	0.55 (0.11 - 2.79)
Reintubation (n = 85)	8/50 (16)	4/35 (11)	0.75†	1.15 (0.74 - 1.82)
Need for tracheotomy‡	4 (8)	11 (25)	0.04†	0.32 (0.11 - 0.93)
Acute renal failure	1 (2)	2 (4)	0.59†	0.61 (0.12 - 3.1)
CRRT	0 (0)	2 (4)	0.21†	NA
Early VAP§	3 (6)	0 (0)	0.24†	NA
Late VAP¶	1 (2)	5 (11)	0.09†	0.29 (0.05 - 1.80)
Bacteremia	2 (4)	1 (2)	1.00†	1.26 (0.55 - 2.88)
Urinary tract infection	1 (2)	5 (11)	0.09†	0.29 (0.05 - 1.80)
Ventilator-free at 28 days	28 (26 - 28)	26 (19 - 28)	0.01	
Total MV|| (days)	5 (2 - 13)	9 (3 - 22)	0.01	
ICU stay (days)	9 (5 - 20)	14 (6 - 29)	0.11	
Hospital stay (days)	24 (17 - 48)	33 (19 - 52)	0.26	
ICU mortality	4 (8)	3 (7)	1.00†	1.08 (0.55 - 2.11)
Hospital mortality	5 (10)	8 (18)	0.37†	0.69 (0.33 - 1.41)
90-d mortality	5 (10)	10 (23)	0.16	0.15 (0.01 - 2.21)

95%CI - 95% confidence interval; NIV - noninvasive ventilation; CRRT - continuous renal replacement therapy; NA - not appropriate; VAP - ventilator-acquired pneumonia; MV - mechanical ventilation; ICU - intensive care unit. Results expressed as n (%). * Cause of extubation failure: Interventional group: neurological deterioration (n = 1), acute respiratory failure (n = 2), excessive tracheobronchial secretions (n = 1), laryngeal stridor (n = 3), atelectasis (n = 2); Control group: excessive tracheobronchial secretions (n = 5), laryngeal stridor (n = 1); † Fisher’s exact test; ‡ Adding tracheotomy before and after extubation. Causes of tracheotomy in the Intervention Group: extubation failure (n = 4). Causes of tracheotomy in the Control Group: low level of consciousness (n = 6), prolonged mechanical ventilation (n = 3) [spontaneous breathing trial failure (n = 1), amount of secretions (n = 2)] and extubation failure (n = 2); § Early ventilator-acquired pneumonia (before 7 days from mechanical ventilation): Intervention Group: *Methicillin-sensitive Staphylococcus aureus* (n = 2), Klebsiella pneumoniae (n = 1); ¶ Late ventilator-acquired pneumonia (after 7 days from mechanical ventilation): Intervention Group: *Stenotrophomonas maltophilia* (n = 1). Control group: *Pseudomonas aeruginosa* (n = 4), *Stenotrophomonas maltophilia* (n = 1)*;* || Total mechanical ventilation was defined as the duration of mechanical ventilation until the ventilator was finally switched off, leading to definitive extubation or disconnection of the patient by tracheotomy, without the need for connection to mechanical ventilation or noninvasive ventilation.

Number of patients who were alive and breathing without invasive mechanical ventilation during the 28 days. Log rank test (p = 0.387).

Table depicts the number of surviving patients during the study period. HR - hazard ratio.

## DISCUSSION

Weaning neurocritical patients from MV is challenging for several reasons: intubation is frequently required due to central neurological damage, which leads to dysfunction of either the ventilatory stimulus or airway control; and these patients are under MV for prolonged periods, which increases the risk of weakened respiratory muscles and respiratory infections.^(12,32)^ Furthermore, a high rate of extubation failure (26%) was observed in a previous retrospective study of 208 patients who had passed an SBT, in which frequent aspiration of secretions was a determinant of extubation failure (odds ratio 5.699; 95%CI 1.863 - 17.432).^(19)^ Thus, we considered that a study in which the implementation of a weaning protocol (Intervention Group) was compared with a conventional protocol (Control Group) in clinical practice would improve the previously described outcomes.^(19)^ The aim of the present study was to comprehensively assess the outcome of early extubation in neurocritical patients who underwent a daily assessment to determine their readiness to wean, ability to breathe spontaneously and ability to maintain a patent airway compared with the usual procedure, where the subjective impression of the physician is the decisive factor in initiating weaning and extubation of the patient. There are serious drawbacks to the study. First, the design of the study without randomization would suggest the possibility of bias. Second, there were changes in the research team that made it necessary to terminate the study. As a consequence, we did not reach the estimated sample size, and we have to assume that the study lacks statistical power to assess the primary objective of the study. Therefore, we have to accept the null hypothesis that a weaning protocol did not reduce extubation failure compared with conventional weaning. However, compared to the Control Group, the Intervention Group had a higher extubation rate, but the duration of MV and the need for tracheotomy were reduced.

The first question of this study is, for neurocritical patients, what is the appropriate level of consciousness to initiate weaning and perform extubation? Unfortunately, it is not easy to determine the appropriate level of consciousness for these patients. In several studies, authors have chosen a GCS > 8 (or a motor score > 4) as a cutoff to consider extubation;^(2,7,18,22,23,33)^ however, in other studies, authors chose the recovery of the neurological disease.^(8,10,34)^ The absence of uniform criteria might cause patients who could be extubated to be excluded from studies or to be directly tracheotomized.^(9,22,34)^ A multicenter observational study compared neurological patients with nonneurological patients and showed that the rate of primary tracheotomy was higher in neurological patients than in nonneurological patients (14% to 29% *versus* 13%, p < 0.001).^(2)^ Similarly, a prospective multicenter international observational study showed a primary tracheotomy rate of 21%. The main cause of tracheotomy was a low level of consciousness (73%).^(35)^ We observed a higher proportion of directly (primarily) tracheotomized patients in the Control Group (20%), mostly due to a low level of consciousness according to physician criteria. Again, physician subjectivity probably influenced decision-making, given that *a priori* of the muscle strength variables analyzed for inclusion in the study indicated that extubation could have been attempted. In conclusion, there is no recommended GCS cutoff to consider the onset of weaning.^(3,4)^ The decision to attempt extubation or tracheotomy is controversial. Despite the possible benefits of tracheotomy (shorter durations of MV and ICU stay), it is recommended that extubation be attempted before performing a tracheotomy.^(4,32)^ Similar to several studies, we considered that patients with a good level of consciousness (GCS > 9 points) would be ready for extubation.^(22,24)^ However, the effectiveness of extubation in patients with a low level of consciousness (GCS ≤ 8) is questioned. A study of a small sample of patients with a low level of consciousness, a successful SBT and an ability to maintain a patent airway (Airway Care Score ≤ 7) showed a low reintubation rate (12.5%) after early extubation.^(36)^ Similarly, in a prospective observational study, 80% of patients with a brain injury with a GCS ≤ 8 (31 patients) and 91% of patients with a GCS ≤ 4 (10 patients) were successfully extubated.^(6)^ Therefore, the applicability of the protocol of the present study in this subgroup of patients is questionable and requires further study.

The second question addressed by this study is, would a protocol that employs three steps (objective assessment of readiness to wean, SBT, and assessment of ability to maintain a patent airway) be effective? First, the use of objective criteria as the first weaning step allowed us to start weaning more quickly, avoiding the subjectivity of physicians.^(20,34)^ Second, we performed an SBT by means of a T-tube as a second weaning step,^(6,9,10,27,28,34,35)^ but in clinical practice, it would not be able to differentiate those patients who will fail, since those patients do not usually have problems that manifest with this test (e.g., heart failure).^(12)^ Moreover, a survey conducted in three North American hospitals studied the extubation criteria for patients who had a successful SBT and showed that in 37% of cases, intensivists delayed extubation.^(20)^ In a multicenter observational study of neurological patients, between 35% and 53% of patients with GCS ≥ 8 and a successful SBT, compared with 55% of nonneurological patients, progressed to weaning.^(2)^ Similarly, a multicenter observational study compared two timings of extubation (before or on the day when the extubation criteria were met *versus* delayed extubation) in neurocritical patients who passed an SBT. The duration of MV and the length of ICU stay were shorter in the early extubation group than in the delayed extubation group: 4 (3 - 5) days *versus* 8 (7 - 12) days; p < 0.01, and 6 (4 - 13) days *versus* 13 (8 - 11) days; p < 0.01, respectively. Furthermore, there were no differences in extubation failure between the two groups (19% prompt extubation *versus* 27% delayed extubation; p = 0.27). The level of consciousness (odds ratio 0.96; 95%CI 0.74 - 1.26) was not related to extubation failure. On the other hand, the level of consciousness was related to delayed extubation (odds ratio 0.30; 95%CI 0.17 - 0.54). ^(34)^ Coplin et al. demonstrated that delayed extubation (due to low GCS) in neurocritical patients capable of being extubated increased the duration of MV, rate of pneumonia, and length of ICU stay, which correlated with higher health care costs.^(6)^ Finally, in a randomized study, only 25% of neurocritical patients who passed an SBT were extubated because of concerns about level of consciousness.^(22)^ These data indicate that the physician’s subjectivity probably delayed extubation in a patient with an acceptable level of consciousness (GCS > 8 points) who had a successful SBT. The level of consciousness is likely to be a limiting factor in progressing to weaning. Third, after a successful SBT, we assessed the ability to maintain airway patency through secretion clearance.^(4,5,15,16,17,22,33)^ In several observational studies of neurocritical patients, copious secretions and weak cough were associated with extubation failure.^(10,33,34,35,37)^ In short, predictive scores for extubation failure have been suggested, which are used to evaluate the level of consciousness and ability to maintain a patent airway.^(9,10,33,35)^ Similarly, a before-after study of the implementation of several ventilatory bundles showed the same results as those that we obtained. The duration of MV was shorter in the study group than in the Control Group (12.6 days *versus* 14.9 days; p = 0.02). In addition, there was no difference in the extubation failure rate (13.5% *versus* 9%; p = 0.11).^(24)^ As a result of following the three steps of the protocol, we observed that the Intervention Group had a higher extubation rate than the Control Group, without any significant difference in the SBT failure and extubation failure rates; however, the duration of MV in the Intervention Group was shorter than that in the Control Group, and the Intervention Group also had a lower rate of tracheotomy, thereby raising questions about the suitability of extubation based on the subjective decision of the physician. However, the subjective decision of the physician probably did not influence the extubation failure rate compared with delayed extubation because all patients met the conditions to be extubated.^(9,22,24,34)^ Surprisingly, the lower rate of extubation failure in the Control Group (17%) compared to our initial results (26%)^(19)^ might be due to the selection of the sample. In the previous study, the patients who failed extubation had a lower level of consciousness (GCS 12 ± 3) and worse respiratory status (RR 24 ± 6 bpm) than in the current study, which could imply an association with a high extubation failure rate.^(19)^ Therefore, a weaning protocol would probably reduce the extubation failure rate in a sample of patients who were not so selective and for whom respiratory strength parameters were not measured.

In the present study, several limitations were identified. First, there could have been biases because the characteristics of the study were not randomized. This had a negative influence on the internal validity of the study. We are aware that there is a certain degree of bias because each physician chose to perform the usual procedure or to use a more demanding protocol. Despite all of the above limitations (absence of randomization and blinding), the homogenization of the sample before inclusion in the study and compliance with the rules that regulate this type of study provide the highest methodological quality.^(25)^ Second, an early conclusion of the study before reaching the sample size (94 instead of 218 patients) due to the loss of part of the research team ostensibly reduces the statistical power of the study and the applicability of the results. Therefore, we must interpret the results cautiously. Third, an earlier inclusion of the cuff leak test in the protocol may have reduced the failure rate, since three patients had a failed extubation due to laryngeal stridor. Despite the moderate quality of the evidence, this test is recommended in patients at risk for laryngeal stridor.^(13)^ Nevertheless, in clinical practice, its use is limited, as reflected in an international survey.^(38)^ Moreover, in neurocritical patients, a cuff leak test has also not been associated with extubation failure.^(34,35)^ Fourth, in the Control Group, a similar extubation failure rate and a lower reintubation rate compared with the Intervention Group could be due to the small sample size obtained, patients who underwent a primary tracheotomy and the use of NIV. Noninvasive ventilation is not recommended in neurocritical patients because copious secretions or neurological deterioration has a negative effect on the effectiveness of NIV.^(39)^ However, a retrospective study reported benefits of NIV compared with tracheotomy in neurocritical patients. In the NIV group, the infection rate and duration of MV were lower than those in the tracheotomized group [54.5% *versus* 84.1%; p = 0.005, and 123 (89.5 - 218.0) hours *versus* 195 (127.3 - 372.3) hours; p = 0.005].^(40)^ In our study, NIV was used on several occasions to avoid reintubation (2% *versus* 6%) despite not being a common procedure in these patients.^(19)^ There are several studies showing that NIV is used in neurocritical patients.^(10,23,35)^ However, there is no recommendation for its use in the established guidelines.^(4)^ Thus, NIV can be indicated if the patient meets the criteria, such as a good level of consciousness or a normal amount of secretions, which are causes of failure in neurocritical patients.^(3,29)^

## CONCLUSION

Considering the limitations of our study, the application of a protocol for neurocritical patients who were evaluated for readiness to wean, ability to breathe spontaneously and ability to maintain airway patency led to a high percentage of extubation, a reduced need for tracheotomy and a shortened duration of mechanical ventilation. However, there was no reduction in extubation failure or the 28-day free of mechanical ventilation compared with the Control Group.
